# Potential Diffusion Tensor Imaging Biomarkers for Elucidating Intra-Individual Age-Related Changes in Cognitive Control and Processing Speed

**DOI:** 10.3389/fnagi.2022.850655

**Published:** 2022-04-26

**Authors:** Shulan Hsieh, Meng-Heng Yang

**Affiliations:** ^1^Cognitive Electrophysiology Laboratory: Control, Aging, Sleep, and Emotion, Department of Psychology, National Cheng Kung University, Tainan, Taiwan; ^2^Institute of Allied Health Sciences, National Cheng Kung University, Tainan, Taiwan; ^3^Department of Public Health, National Cheng Kung University, Tainan, Taiwan

**Keywords:** white matter integrity, DTI, FA, MD, RD, AxD, cognitive control, processing speed

## Abstract

Cognitive aging, especially cognitive control, and processing speed aging have been well-documented in the literature. Most of the evidence was reported based on cross-sectional data, in which inter-individual age effects were shown. However, there have been some studies pointing out the possibility of overlooking intra-individual changes in cognitive aging. To systematically examine whether age-related differences and age-related changes might yield distinctive patterns, this study directly compared cognitive control function and processing speed between different cohorts versus follow-up changes across the adult lifespan. Moreover, considering that cognitive aging has been attributed to brain disconnection in white matter (WM) integrity, this study focused on WM integrity via acquiring diffusion-weighted imaging data with an MRI instrument that are further fitted to a diffusion tensor model (i.e., DTI) to detect water diffusion directionality (i.e., fractional anisotropy, FA; mean diffusivity, MD; radial diffusivity, RD; axial diffusivity, AxD). Following data preprocessing, 114 participants remained for further analyses in which they completed the two follow-up sessions (with a range of 1–2 years) containing a series of neuropsychology instruments and computerized cognitive control tasks. The results show that many significant correlations between age and cognitive control functions originally shown on cross-sectional data no longer exist on the longitudinal data. The current longitudinal data show that MD, RD, and AxD (especially in the association fibers of anterior thalamic radiation) are more strongly correlated to follow-up aging processes, suggesting that axonal/myelin damage is a more robust phenomenon for observing intra-individual aging processes. Moreover, processing speed appears to be the most prominent cognitive function to reflect DTI-related ***age*** (cross-sectional) and ***aging*** (longitudinal) effects. Finally, converging the results from regression analyses and mediation models, MD, RD, and AxD appear to be the representative DTI measures to reveal age-related changes in processing speed. To conclude, the current results provide new insights to which indicator of WM integrity and which type of cognitive changes are most representative (i.e., potentially to be neuroimaging biomarkers) to reflect intra-individual cognitive aging processes.

## Introduction

Cognitive aging has been extensively investigated over the past two decades. One of the critical reasons for this research trend is due to the crisis of population aging which encourages researchers paying attention to evaluate the trajectories of cognitive function decline across chronical age to help people age gracefully. One pronounced diagram showing behavioral performance on measures of processing speed, working memory, long-term memory, and world knowledge was first reported by Park et al. (2002), in which while many cognitive functions, such as speed, spatial orientation, problem solving, numerical ability, verbal memory were significantly declined with age, whereas verbal ability (e.g., vocabulary) did not show decline with age. However, these trajectories of functional decline with age was based on the cross-sectional aging data (Light, 1991; Park, 2002; Park et al., 2002; Salthouse, 2004), it is still unclear whether similar results could also be seen in longitudinal (or follow-up) data. Literature has highlighted an important distinction between ***age*** effects and ***aging*** effects (see Rugg, 2017). An ***age*** effect refers to a scenario in which a dependent variable differs between groups of individuals with different mean ages (such as contrasting a younger group with an older group)—this is known as a cross-sectional design. Whereas an ***aging*** effect refers to a scenario in which we observe an individual’s performance changes over time—this is known as a longitudinal or follow-up design. Rugg (2017) has indicated several inferring limitations regarding age-related cognitive aging using a cross-sectional design. Some studies have also provided evidence in showing discrepant results coming from cross-sectional versus longitudinal data, hence highlighting the importance of longitudinal evidence to reveal actual aging effect in cognition (Hedden and Gabrieli, 2004; Hsieh and Yang, 2021).

### Brain Structural Connectivity and Cognitive Control in Age/Aging

Cognitive aging has been attributed to brain disconnection in white matter integrity, in which a disruption of communication between cortical regions can result in cognitive dysfunction (O’Sullivan et al., 2001; Bartzokis, 2004; Andrews-Hanna et al., 2007; Fjell et al., 2016; Madden et al., 2017). For human studies, white matter (WM) integrity can be measured via acquiring diffusion-weighted imaging (DWI) data with a magnetic resonance imaging (MRI) instrument that are further fitted to a diffusion tensor model (i.e., diffusion tensor imaging; DTI) to detect water diffusion directionality, which in turn shows the microstructural architecture of tissue. Through DTI fitting model (Behrens et al., 2003), one can derive some indicators reflecting the degree of tissue integrity, such as fractional anisotropy (FA), mean diffusivity (MD), radial diffusivity (RD), and axial diffusivity (AxD). DTI can detect microstructural WM abnormalities preceding the lesions (de Groot et al., 2013; Maillard et al., 2014), and further reveal the neurobiological mechanism(s) of axonal fiber damage (Le Bihan et al., 2001; Vernooij et al., 2009). Specifically, it has been shown that lower FA and higher MD indicate the overall reduction in WM fiber integrity (Le Bihan et al., 2001), and elevated RD and AxD may, at least in part, reflect axonal demyelination and/or degeneration (Song et al., 2003; Klawiter et al., 2011). By means of these indicators, literature has shown a decrease in FA and increase in MD with increasing age, suggesting a decreased WM integrity with age (Salat et al., 2005; Yap et al., 2013; Lebel et al., 2012; Sexton et al., 2014; de Groot et al., 2015; de Lange et al., 2016; Marques et al., 2016). In addition, literature has also shown a significant relationship between WM integrity and cognitive performance in older adults (Bennett and Madden, 2014; see Madden et al., 2012 for a review). Although these previous studies have reported the association between WM integrity and age, and between WM integrity and cognitive performance, most studies used a cross-sectional design and did not provide direct evidence showing whether WM integrity plays a mediation role in the association between age/aging and cognitive performance. Furthermore, some existing longitudinal studies focused on specific WM tracts rather than a whole brain WM integrity. Therefore, the primary aim of this study was to fill the research gap by incorporating both cross-sectional (***age***) and follow-up (***aging***) designs to examine the relationship among age/aging, WM integrity measured by DTI metrics (e.g., FA, RD, MD, and AxD), and cognitive performance. We hypothesized that the results for age-related differences might be different from age-related changes due to cohort and other factors (Hedden and Gabrieli, 2004; Rugg, 2017; Hsieh and Yang, 2021).

For cognitive performance, in this study, we focused on processing speed and cognitive control abilities. This is because the prominent cognitive aging manifests in general slowing (Madden and Allen, 1995) and cognitive control dysfunction (Craik and Byrd, 1982). Furthermore, the age-related deterioration of WM integrity has been observed to be particularly vulnerable to frontal regions (Davis et al., 2009; Bennett et al., 2010; Burzynska et al., 2010). Therefore, we hypothesized that WM integrity should be related to age-related cognitive control changes. To directly test this hypothesis, we also employed a mediation model for longitudinal data to see if WM integrity mediates age-related changes in processing speed and/or cognitive control. Cognitive control function is a broad term. In this study, we adapted the definition by Miyake et al. (2000) which suggests components of inhibition (e.g., measured by a stop-signal task; to note, the inhibition component was later-on modified as a common component), updating (e.g., working memory measured by a n-back task), and shifting (e.g., measured by a paper-and-pencil Trail Making Test (TMT) and a computerized task-switching paradigm) components. As for measuring processing speed, we used a commonly used neuropsychological test, Grooved Pegboard Test (GPT) to collect visuo-motor action speed. In addition, we derived some basic processing speed indicators from TMT form A, and from some computerized cognitive control task in which the basic processing conditions are included (e.g., go trials’ reaction time in a stop-signal task; repeat trials’ reaction time in a task-switching paradigm). We used both original task’s performance indexes and the transformed indexes suggested by Miyake et al. (2000), please see detail in the “Materials and Methods” Section.

Using the definitions of cognitive control based on Miyake’s model, some specific issues could be addressed in this study. First, regarding the association between age/aging and cognitive control (including processing speed), we would like to know what type of cognitive control functions are more closely related to age/aging. Second, regarding the association between age/aging and DTI measures, we would like to examine which WM tracts and which WM integrity’s indicators (i.e., FA, MD, RD, and AxD) would be more closely related to age/aging. Third, regarding the association between cognitive control functions (including processing speed) and DTI measures, we would like to examine which cognitive performance is more closely related to which WM integrity’s indicator. Fourth, whether age-related changes in WM integrity would mediate the association between aging and cognitive performance. Since these four issues have not been directly explored previously, we did not set up any specific predictions, but simply hypothesized that there should be different patterns among different types of cognitive control functions and among different DTI metrics with age/aging effects.

## Materials and Methods

### Participants

We used advertisements on the Internet and bulletin boards to recruit hundreds of right-handed participants from southern Taiwan. Participants’ medical information including neurological history and mental health status were collected via a self-report. The participants included in this study all reported no history of any psychiatric or neurological disorders and they also past the screening criteria of the two neuropsychological tests, including the Montreal Cognitive Assessment (MoCA) to screen for cognitive impairment if the scores were ≤25 (Nasreddine et al., 2005; Chinese Version: Tsai et al., 2012), and the Beck Depression Inventory-II (BDI-II; Beck et al., 1996; Chinese version published by Chinese Behavioral Science Corporation) to screen for depression if the scores were ≥14. A total of 121 qualified participants completed the two follow-up sessions (with a range of 1–2 years). In each session, participants completed the questionnaire of demographic information, computerized cognitive tasks, neuropsychological tests for measuring cognitive control and processing speed, and magnetic resonance imaging (MRI) acquisitions. Four participants were excluded because of technical problems with MRI or incomplete data. Three participants were further excluded because their DTI’s measures >5 standard deviations. Furthermore, all remaining 114 participants’ images passed quality control. We also visually inspected all images after normalization and co-registration steps. This ensures that there is no serious warping. The mean age of the remaining 114 participants (females’ ratio = 60.53%) was 48.72 ± 16.54 years [timepoint 1 (TP1); 20.25–77.92 years] and 50.49 ± 16.64 years [timepoint 2 (TP2); range 21.92–79.83 years]. See Table 1 for the participants’ age range distribution and demographic information.

**TABLE 1:** Participants’ demographic information and DTI measures for time point 1 (TP1) and time point 2 (TP2) and their corresponding paired *t*-tests.

	TP1	TP2	Paired *t*-test (*p-values*)
*N*	114	114	N/A
Age	48.72 (±16.54) range: 20.25∼77.92	50.49 (±16.64) range: 21.92∼79.83	**0.000**
Gender (F%)	60.53%	60.53%	N/A
Education (Y)	15.02 (±2.65)	15.02 (±2.65)	N/A
BDI-II	4.97 (±4.18)	5.39 (±6.06)	0.440
MoCA	27.63 (±1.86)	28.97 (±1.25)	**0.000**
FA	0.4387 (±0.01593)	0.4389 (±0.01606)	0.624
MD	0.760*10^–3^ (±0.022*10^–3^)	0.763*10^–3^ (±0.023*10^–3^)	**0.000**
RD	0.560*10^–3^ (±0.025*10^–3^)	0.563*10^–3^ (±0.026*10^–3^)	**0.003**
AxD	1.159*10^–3^ (±0.020*10^–3^)	1.166*10^–3^ (±0.021*10^–3^)	**0.000**

*p-values marked in bold are those also passing the Bonferroni correction method. BDI-II, Beck Depression Inventory; MoCA, Montreal Cognitive Assessment; FA, fractional anisotropy; MD, mean diffusivity; RD, radial diffusivity; AxD, axial diffusivity.*

The study was carried out in accordance with the Declaration of Helsinki and the study protocol was approved by Ethical Committee at the National Cheng Kung University (reference number #104-004). Participants received monetary compensation for their participation after the completion of all assessments (NTD$1,500 per session).

### Computerized Cognitive Tasks for Measuring Cognitive Control Performance and Processing Speed

#### General Instruments for Visual Presentation

The visual stimuli used in the following computerized tasks were programmed using Presentation software, and were displayed on a 17-inch monitor with 1024*768 resolution.

##### Stop-signal task

The stop-signal task was a modified version of Logan’s paradigm (Logan et al., 2014). Participants were asked to fixate at the visual stimulus on the screen and use their index fingers of both hands to press either the “z” or “/” stroke on the keyboard when target “O” or “X” was presented. Participants were told to react to the stimulus as quickly and accurately as possible. On some occasions, a “beep” sound with a duration of 100 ms which served as a “stop” signal might be delivered following a target with a delay (i.e., stop-signal delay; SSD) initially set at 150 or 350 ms, and adjusted with a staircase tracking procedure (i.e., decreased by 50 ms following a failure stop and increased by 50 ms following a successful stop). Participants were informed to ignore this sound in the first practice session, so that they could be familiar with quickly responding to the stimuli. In the second practice session, participants were told to immediately stop their intended action once they heard the “beep” sound. We reminded participants with the instruction of “Do not hold your responses while waiting for a beep sound.” Following the two practice sessions, there were four experimental blocks which contained randomly intermixed 40 stop trials (i.e., trials followed by a stop sound) and 100 go trials without a stop signal. The whole duration for this task was about half an hour.

The stop-signal reaction time (SSRT) indexing the stopping efficiency was calculated by subtracting the median SSD from the median RT of the go trials. The larger the SSRT indicates the worse stopping efficiency.

##### Task-switching paradigm

Task-switching abilities were measured by a modified paradigm from Karayanidis et al.’s (2003) study. Each trial contained a cue (non-informative or informative) with a duration of 600 ms, and followed by a target display with an interval of 1,000 ms (i.e., cue-target interval, CTI). An informative cue was a colored cross with either a warm color (red or orange) or a cold color (green or blue) indicating which is the forthcoming task: a letter classification or a number classification task. A non-informative cue was colored in gray, which provided no information about the forthcoming task type. The target display contained a pair of two stimuli, in which there were two different pair types: an incongruent pair and a neutral pair (50% each in a mixed task block). An incongruent pair contained a Chinese letter and an Arabic number, and a neutral pair only contained one of them and paired with a symbol sign (e.g.,%, #, @, or $). The Chinese letter was one of eight Chinese letters which were retrieved from the Ten Celestial Stem system (i.e., Tiangan). Participants were asked to respond to the stimulus display using either their right or left index finger that was mapped to the first-half/second-half or odd/even for Chinese-letter and Arabic-number tasks, respectively.

All participants practiced six blocks before the experiment. The experiment contained 12 blocks: (1) two single-task blocks which contained only Arabic numbers (i.e., a neutral pair) for a sequence of 70 trials per block; (2) two single-task blocks which contained only Chinese letters (i.e., a neutral pair) for a sequence of 70 trials per block; (3) four mixed informative task blocks of 70 trials per block (with a switch rate around 33.3%); and (4) four mixed non-informative task blocks of 70 trials per block (with a switch rate around 33.3%). The entire experiment lasted for about 35∼40 min.

We calculated the switch cost by subtracting the average RT of the repeat trials in the mixed-task blocks from the average RT of the switch trials in the mixed-task blocks.

##### Spatial n-back task

In this study, we adapted a spatial version of 1-back and 2-back tasks to measure working memory updating function. The spatial n-back is a single n-back task based on spatial locations as the stimuli stream. For example, in this study, the stimuli were presented within a 3-by-3 grid in each trial. One of the grid squares was randomly assigned to be filled with blue. In a 1-back task, participants were asked to continuously memorize the position of the blue grid square shown in the previous trial and to match it to the current trial’s position of the blue grid square. In a 2-back task, participants were asked to continuously memorize the position of the blue grid square in the previous two trials and to compare it to the current trial’s position of the blue grid square. If the blue grid square appeared in the same location as instructed (either matched to the previous one trial’s grid position in a 1-back task or the previous two trial’s grid position in a 2-back task), participants pressed the “F” button using their left index finger. If the blue grid square appeared in a different location, participants pressed the “J” button using their right index finger. Each grid stimulus appeared for 500 ms followed by an interstimulus interval (ISI) of 2,000 ms. Participants completed one practice block with performance feedback, and three experimental blocks which contained 21 trials per block. This entire experiment lasted for 30 to 40 min.

We calculated performance sensitivity (*d*′) as a working memory updating index, which was based on the hit rate (H) and false-alarm (F) rate. We first transformed the raw data into *z* scores. The formula is as follows: *d*′ = *Z*(H) − *Z*(F) (*Z* denotes the *z* score of the normal distribution). The larger values of the *d*′ indicates higher working memory updating ability. Therefore, the direction of the *d*′ value is the opposite to the other indexes, such as inhibition and shifting. Therefore, to equalize the direction of the performance for these three types of cognitive control indexes, we transformed *d*′ into negative values, so that the higher *d*′ value (i.e., less negative) would indicate worse updating performance which yields a similar performance direction for SSRT, switch costs, and *d*′.

#### Computing Common and Specific Executive Function Components – Miyake’s Definition

The performance on each of the three tasks (switch cost in a task-switching paradigm, SSRT in a stop-signal task, and *d*′ in a n-back task) were calculated into *z* value. Following Miyake and Friedman’s procedures, we averaged these three *z* scores to make a composite score reflecting common EF for every participant. We regressed SSRT and n-back *d*′, and used the residuals as a shifting EF component. We regressed *d*′ against SSRT and switching cost, and used the residuals as an updating EF component.

### Neuropsychological Tests for Measuring Cognitive Control Performance and Processing Speed

#### Trail Making Test

In this study, we used the Chinese version of the Trail Making Test (TMT), which consisted of two forms (A and B) of task conditions. The reliability of the Chinese version of the TMT has been reported by Wang et al. (2018). Form A consisted of numbers from “1” to “25” and was displayed randomly on an A4 sheet of paper. Form B consisted of digit numbers from “1” to “12” and Chinese zodiac letters (“rat” to “pig”) were shown. Participants drew a line connecting these items during a sequence of “1”–“2”–“3”–…“25” in form A and an alternating sequence of “1”–“rat”–“2”–“ox”–…“12”–“pig” in form B. The time to finish the form (TMT-A, TMT-B) was recorded as a performance index. TMT-A is considered as a metric reflecting processing speed, while TMT-B is a metric of switching proficiency plus processing speed.

#### Grooved Pegboard Test

Participants were asked to insert cylindrical metal pegs into 25 holes of a pegboard as fast as possible. Left- and right-handed performances were tested separately. The test began with the self-identified dominant hand (i.e., the right hand in this study), followed by the non-dominant hand. Participants were asked to insert pegs in the standardized order (from left to right for all rows when using the right hand and from right to left for all rows when using the left hand) and to use just one hand at a time. The whole time to finish the test was recorded as an index of processing speed for each hand, respectively (GPT_R; GPT_L).

### Cognitive Performance Indexes for Cognitive Control and Processing Speed

The performance indexes (all transformed into *z* scores) collected from the above series of cognitive tasks and neuropsychological tests are summarized in Table 2, in which TMT-A, GPT_R/L, go RT in a stop-signal task are considered as indexes for processing speed, switch cost (informative and non-informative), TMT-B is considered as indexes of shifting, SSRT is an inhibition index, whereas mixing cost derived from task-switching paradigm, and 1-/2-back *d*’ are considered as indexes of working memory. In addition, as aforementioned, we also calculated common and specific EF components (i.e., common, shifting, updating) derived from the three computerized cognitive control tasks based on Miyake’s model (see Table 2).

**TABLE 2:** The significant Pearson correlation *r* values for age (cross-sectional) and aging (longitudinal) effects in percentage changes of cognitive control and processing speed.

	Cross-sectional cognitive performance (TP1) correlation with age (TP1)	Longitudinal Δ cognitive performance correlation with age (TP1)	Domain
TMT-A	**0.544**	–	Speed
TMT-B	**0.384**	–	Shift
GPT_L	**0.560**	**0.288**	Speed
GPT_R	**0.494**	–	Speed
SSRT	**0.312**	–	Inhibition
goRT	**0.369**	–	Speed
infSWIcost	−0.167	–	Shift
non-infSWIcost	–	–	Shift
MIXcost	–	–	WM updating
2-back *d*′	**0.367**	–	WM updating
1-back *d*′	–	–	WM updating
Common EF	**0.272**	–	Inhibition
Shifting EF	0.156	–	Shift
Updating EF	**0.257**	–	WM

*The r value denoted in the table represents its p value passing the bootstrap criteria (i.e., upper and lower bound do not pass 0). r value marked in bold indicates its p value also passing the Bonferroni correction criteria (p < 0.0004). ‘–’ denotes non-significance. TMT, Trail Making Test; GPT, Grooved Pegboard Test (L, left hand; R, right hand); SSRT, stop signal reaction time; infSWIcost, inform condition’s switch cost; non-infSWIcost, non-inform condition’s switch cost; MIXcost, mixing cost; EF, executive function; WM, working memory.*

### Neuroimaging Acquisition and Analysis

#### Image Acquisition

All brain images in this research were acquired using a GE MR750 3T scanner (GE Healthcare, Waukesha, WI, United States) installed in the Mind Research and Imaging Center at National Cheng Kung University (NCKU).

High-spatial-resolution T1-weighted images were acquired with fast spoiled gradient echo (fast-SPGR) (TR/TE: 7.6 ms/3.3 ms; flip angle: 12°; FOV: 22.4*22.4 cm^2^; thickness: 1 mm; matrices: 224*224). A total of 166 axial slices was acquired during a scan time of 218 s.

Diffusion tensor imaging was acquired using a spin-echo echo-planar imaging sequence with the acquisition parameters: TR/TE = 5500 ms/62∼64 ms, 50 directions with *b* = 1000 s/mm^2^, 100 × 100 matrices, slice thickness = 2.5 mm, voxel size = 2.5 × 2.5 × 2.5 mm, number of slices = 50, FOV = 25 cm, NEX = 3. The total scan time for the DTI acquisition was 924 seconds. A reversed-phase-encoding DTI was also acquired for off-line top-up corrections in the DTI preprocessing. The acquisition parameters for the reversed-phase-encoding DTI were identical to the DTI, with the only difference being the number of directions as six. The total scan time for the reversed-phase-encoding DTI was 198 seconds. The reason for choosing fewer numbers of reversed-phase-encoded directions was to avoid motion artifacts due to long scanning times.

#### Diffusion Tensor Imaging Processing

We used the FMRIB software Library (FSL v5.0.9^1^; Smith et al., 2004) for all diffusion-weighted imaging (DWI) data processing.

The preprocessing steps were identical to those of earlier work (for details, refer to Yang et al., 2019). DWI data processing as follows: (1) estimating and correcting susceptibility induced distortions using “*topup*” tool, (2) correcting slice-to-volume movement using “*eddy_correct*,” (3) fitting a diffusion tensor model to the images using “*dtifit*” to obtain scalar DTI maps, in which each voxel was assigned with three eigenvalue (principal diffusivities: λ1, λ2, λ3) and three eigenvector (principal directions: v1, v2, v3), describing the water diffusion within the voxel., and (4) performing voxel-wise statistical analyses of the fractional anisotropy (FA) data by using tract-based spatial statistics (*TBSS*; Scahill et al., 2003) to register and normalize all participants’ FA images to the MNI standard space. This process was then repeated for MD and RD images using the tbss_non_FA function.

Subsequently, for tract-of-interest (TOI) analyses, TBSS-skeleton binary masks were overlaid with atlas binary masks which were created with a threshold of 5% based on the probabilistic Johns Hopkins University (JHU) white-matter tractography atlas in FSL (provided by the ICBM DTI workgroup). We chose anterior thalamic radiation (ATR) left (L) and right (R) hemisphere, cingulum/cingulate gyrus (CG) L/R, cingulum/hippocampus (CH) L/R, corticospinal tract (CST) L/R, forceps major (Fmaj), forceps minor (Fmin), inferior fronto-occipital fasciculus (IFF) L/R, inferior longitudinal fasciculus (ILF) L/R, superior longitudinal fasciculus (SLF) L/R, and uncinate fasciculus (UF) L/R as TOIs. Fmin is the commissural fibers of the anterior corpus callosum, whereas Fmaj is the commissural fibers of the posterior corpus callosum. Then we used these masks to mask the FA/MD/RD/AxD map which produced from the TBSS step for each participant. The average FA/MD/RD/AxD values were computed and used in the following analysis.

### Statistical Analyses

To evaluate the relationship between age, FA/MD/RD/AxD and cognitive performance. In the first part, we tested cross-sectional correlations between (1) age and cognitive performance, (2) age and FA/MD/RD/AxD, and (3) cognitive performance and FA/MD/RD/AxD. For all these three sets of Pearson *r* correlation analyses, we used gender, education and BDI-II as covariates. In addition, for cross-sectional data, we also tested the quadratic effects of age (i.e., age^2^).

In the second part, we used longitudinal data to calculate changes in DTI measures and changes in cognitive performance by subtracting DTI (or cognitive performance score) at time 1 (TP1) from time 2 (TP2) and dividing by the exact number of years between scans adjusted to 2 years. In addition, we considered participants’ baseline scores of TP1 by adding a denominator of TP1 measure into the formula to form percentage change scores (e.g., Simmonds et al., 2014 on early life changes). Furthermore, the time between scans was adjusted to 2 years as the average time between the two acquisitions for the cohort was 1.77 years (e.g., Breukelaar et al., 2017).

The formula is shown below:


$$\begin{array}{c} \left[\left({\mathrm{Measure}}_{\mathrm{TP2}}-{\mathrm{Measure}}_{\mathrm{TP1}}\right)/{\mathrm{Measure}}_{\mathrm{TP1}}\right]\\ \text{x}\left(\frac{2}{TP2-TP1}\right)=\Delta \text{Measure} \end{array}$$

Please note, percentage change scores (i.e., ratio scores) will be denoted as ΔMeasure for brevity throughout the manuscript. We tested longitudinal correlations between (1) age (at TP1) and Δcognitive performance, (2) age (at TP1) and ΔFA/MD/RD/AxD, and (3) Δcognitive performance and ΔFA/MD/RD/AxD. We used gender, education and BDI-II as covariates in all Pearson *r* correlation analyses. All data were transformed into *z* score before analyses in this study.

All analyses with multiple comparisons were corrected using the Bootstrap method in which we ran 1,000 iterations to calculate the bias-corrected and accelerated (BCa) bootstrap interval (upper and lower bond). We also used a conventional Bonferroni correction method for multiple comparisons, that is, we used a critical value of *r* > 0.271, *p* < 0.004 for examining significant correlations of cognitive performance in relation to age, and a critical value of *r* > 0.279, *p* < 0.003 for DTI in relation to age and to cognitive performance. However, here we interpreted the results based on the Bootstrap method because it has the advantage of taking into account the dependence structure of *p* values (Vilar-Fernández et al., 2007).

### Mediation Analysis

For the mediation analysis, we used Mplus version 8 to build a mediation path model with latent DTI variables. The latent DTI variables (FA, MD, RD, AxD separately) were defined by the tract which was significantly correlated with cognitive measurement. This estimated both the direct and indirect effects on all cognitive measurement. The model was estimated using maximum likelihood estimation. The significance of indirect effects was assessed with a 95% confidence interval calculated by the Bootstrap method. To estimate confidence intervals, we used a bias-corrected method with the percentile bootstrap estimation approach, which ran 5,000 bootstrap iterations that were implemented. We rejected the null hypothesis if the interval didn’t include zero.

## Results

Participants’ demographic information and DTI measures for TP1 and TP2 and their statistical tests are shown in Table 1. The results show that age, MoCA, MD, RD, and AxD, but not BDI-II and FA scores, are significantly different between TP1 and TP2. Please note, for each of the cognitive performance index and for each WM tract of the DTI measures between TP1 and TP2, the mean scores and respective paired *t*-tests results are shown in the Supplementary Tables 1, 2.

To address the issues set out in this study, we report the results as follows:

### Associations Between Age/Aging and Cognitive Control (Including Processing Speed)

***Cross-sectional*** result (***age*** effect): Table 2’s left column shows linear correlation between age and cognitive performance at TP1. It can be found that age was significant correlated with processing speed (include TMT-A, GPT_L, GPT_R, and goRT), inhibition (SSRT, common EF), shifting (TMT-B, infSWIcost, and shifting EF component), and updating (2-back *d*′, updating EF component).

***Longitudinal*** result (***aging*** effect): Table 2’s right column shows correlation between age (at TP1) and follow-up percentage changes in cognitive performance. There was only one significance remaining on the follow-up data, i.e., between age (at TP1) and speed changes (ΔGPT_L).

### Associations Between Age/Aging and Diffusion Tensor Imaging

***Cross-sectional*** result (***age*** effect): Table 3’s left columns show Pearson correlation *r* values between age and DTI (FA, MD, RD, and AxD) at TP1. The results show that FA, MD, and RD were all correlated with the age effect, except a few WM tracts such as CG_R, CH_L/R, CST_R in FA, and CG_R, CH_L in MD/RD. Whereas AxD showed fewer WM tracts significantly correlated with the age effect, that is, only ATR_L/R, CH_R, and Fmin were significantly correlated with age at TP1.

**TABLE 3:** The significant Pearson correlation r values for age and DTI (cross-sectional and longitudinal ratio scores).

	Cross-sectional DTI (TP1) correlation with age				Longitudinal DTI correlation with age			
	FA	MD	RD	AxD	FA	MD	RD	AxD
**Association fiber**								
ATR_L	**−0.504**	**0.562**	**0.600**	**0.409**	–	**0.293**	0.251	**0.319**
ATR_R	**−0.474**	**0.570**	**0.599**	**0.434**	–	**0.284**	0.242	**0.344**
CG_L	**−0.436**	0.219	**0.349**	–	–	**0.318**	0.231	**0.377**
CG_R	–	–	–	–	–	–	–	–
CH_L	–	–	–	–	0.199	0.213	–	0.271
CH_R	–	0.267	0.238	0.238	0.193	–	–	–
IFF_L	**−0.531**	**0.319**	**0.430**	–	–	**0.295**	0.224	**0.344**
IFF_R	**−0.477**	**0.323**	**0.407**	–	–	**0.353**	0.251	**0.418**
ILF_L	**−0.481**	0.271	**0.373**	–	–	0.199		0.230
ILF_R	**−0.480**	0.251	**0.360**	–	–	**0.336**	0.243	**0.398**
SLF_L	**−0.429**	0.219	**0.324**	–	–	0.207		0.251
SLF_R	**−0.421**	0.223	**0.321**	–	–	0.256	0.194	**0.314**
UF_L	**−0.450**	0.258	**0.354**	–	–	**0.327**	**0.279**	**0.377**
UF_R	**−0.352**	0.204	**0.285**	–	–	**0.403**	**0.340**	**0.453**
**Projection fiber**								
CST_L	–0.248	0.195	0.255	–	0.215	–	–	0.209
CST_R	–	0.212	0.238	–	–	–	–	0.255
**Commissural fiber**								
Fmaj	**−0.507**	**0.285**	**0.430**	–	–	–	–	–
Fmin	**−0.637**	0.255	**0.465**	**−0.280**	–	–	–	–

*The r value denoted in the table represents its p value passing the bootstrap criteria (i.e., upper and lower bound do not pass 0).; r value marked in **bold** indicates its p value also passing the Bonferroni correction criteria (p < .0003). ‘–’ denotes non-significance.*

**FIGURE 1:**
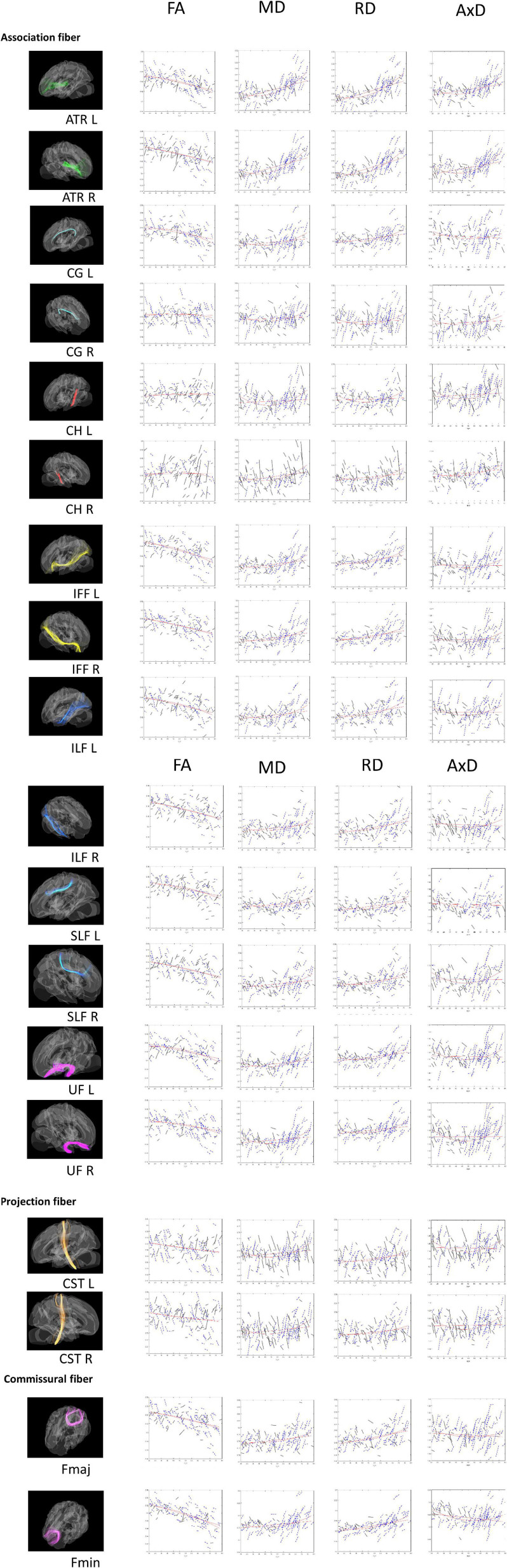
Tract-of-Interest for DTI (FA, MD, RD, and AxD) and their relationships with age. The spaghetti plot that connects the repeated measurements for time point 1 (TP1) and time point 2 (TP2). Tract-of-Interest for DTI measures (FA, MD, RD, and AxD) and their relationships with age is shown in the figure, respectively. Black solid lines denote the better white matter integrity for TP2 than TP1. Conversely, blue dashed lines denote worse white matter integrity for TP2 than TP1. Red lines denote best fitting linear and non-linear regression lines for cross-sectional data on TP1.

As mentioned, we also ran a set of non-linear analyses for the age effect on DTI (see Figure 1). Most of the WM tracts in the quadratic age effects retained the same patterns as those for the linear age effect. *r*^2^ values in CG_L, Fmin, IFF, ILF, SLF and UF remained to be between 0.07 and 0.26. We found that MD and RD had the highest quadratic age effect in ATR L/R (*r*^2^ > 0.4), whereas FA had the highest quadratic age effect in Fmin (*r*^2^ > 0.4). As similar as the linear age effect, the quadratic age effects of FA in CG_R, CH, and CST were not significant (*r*^2^ < 0.03), yet although the indicator of MD for CH_L tract didn’t have a linear effect, it does have a quadratic effect (*r*^2^ = 0.115). Conversely, compared to the linear correlation results, AxD now showed many more WM tracts (except CG, ILF_R, Fmaj) that were significantly associated with age, suggesting that AxD is better interpreted non-linearly for the cross-sectional data.

***Longitudinal*** result (***aging*** effect): Table 3’s right columns show correlations between age (at TP1) and DTI percentage changes from TP1 to TP2 (i.e., ΔFA, ΔMD, ΔRD, ΔAxD). We found that although at cross-sectional data showing FA, MD, and RD were mostly correlated with the age effect, but in the longitudinal data, FA changes (ΔFA) paradoxically showed very few WM tract’s significances with age (at TP1) except that CH_L/R and CST_L tracts were significant. Conversely, although AxD showed fewer WM tracts significantly linearly correlated with age cross-sectionally, its changes (ΔAxD) on many WM tracts showed significantly correlated with age (at TP1), suggesting that AxD index is closely related to intra-individual aging changes. As for MD and RD changes (i.e., ΔMD and ΔRD), they were mostly still significantly correlated with age (at TP1) except a few tracts (e.g., ΔMD: CG_R, CH_R, CST_L/R, Fmaj, & Fmin; ΔRD: CG_R, CH_L/R, ILF_L, SLF_L, CST_L/R, Fmaj, Fmin; see Table 3 for details), suggesting that MD and RD indexes are more representative for both ***age*** and ***aging*** effects, whereas FA seems to be only sensitive to the age effect (i.e., cross-sectional), but not aging effect.

### Cognitive Control (Including Processing Speed) and Diffusion Tensor Imaging

#### Processing Speed and Diffusion Tensor Imaging

***Cross-sectional*** result (***age*** effect): Table 4 left columns show significant correlations between processing speed and FA/MD/RD/AxD measures for the cross-sectional data (TP1). We can find there were many significant correlations between processing speed and DTI measures, with FA being as negative correlations, and MD and RD being as positive correlations. AxD showed relatively fewer significant positive correlations in WM tracts (only in ATR_L/R and CH_L tracts) with processing speed.

**TABLE 4:** The significant Pearson correlation *r* values for processing speed in relation to DTI measures (FA, MD, RD, and AxD) for cross-sectional and longitudinal ratio scores.

	Behavior (covariate: gender, edu, BDI-II)							
	Cross-sectional and processing speed				Longitudinal and processing speed			
	FA	MD	RD	AxD	FA	MD	RD	AxD
**Association fiber**								
ATR_L	**−0.400 (TMT-A)** **−0.337 (GPT_L)** **−0.381 (GPT_R)**	**0.442 (TMT-A)** **0.371 (GPT_L)** **0.318 (GPT_R)** 0.223 (goRT)	**0.464 (TMT-A)** **0.394 (GPT_L)** **0.361 (GPT_R)** 0.225 (goRT)	**0.342 (TMT-A)** 0.277 (GPT_L) 0.199 (goRT)	−0.274 (GPT_R)	–	–	–
ATR_R	**−0.348 (TMT-A)** **−0.311 (GPT_L)** **−0.364 (GPT_R)**	**0.418 (TMT-A)** **0.375 (GPT_L)** **0.345 (GPT_R)** 0.241 (goRT)	**0.430 (TMT-A)** **0.394 (GPT_L)** **0.375 (GPT_R)** 0.230 (goRT)	**0.346 (TMT-A)** **0.292 (GPT_L)** 0.243 (GPT_R) 0.228 (goRT)	–	–	–	–
CG_L	**−0.341 (TMT-A)** **−0.294 (GPT_L)** **−0.291 (GPT_R)**	0.220 (TMT-A) 0.221 (GPT_R)	**0.296 (TMT-A)** 0.263 (GPT_L) **0.279 (GPT_R)**	–	−0.230 (GPT_R)	0.213 (GPT_L)	0.235 (GPT_R)	–
CG_R	–	–	–	–	−0.216 (GPT_R)	–	–	–
CH_L	–	0.263 (TMT-A)	0.222 (TMT-A)	0.263 (TMT-A)	–	**0.304 (GPT_L)**	–	**0.312 (GPT_L)**
CH_R	–	0.270 (TMT-A)	0.277 (TMT-A) 0.175 (GPT_R)	–	–	–	–	–
IFF_L	**−0.385 (TMT-A)** **−0.346 (GPT_L)** **−0.375 (GPT_R)**	**0.315 (TMT-A)** 0.277 (GPT_L) **0.307 (GPT_R)**	**0.365 (TMT-A)** **0.322 (GPT_L)** **0.357 (GPT_R)**	–	**−0.284 (GPT_R)**	0.199 (GPT_R)	0.242 (GPT_R)	0.236 (GPT_L)
IFF_R	**−0.357 (TMT-A)** **−0.314 (GPT_L)** **−0.363 (GPT_R)**	**0.329 (TMT-A)** 0.251 (GPT_L) **0.307 (GPT_R)**	**0.362 (TMT-A)** **0.295 (GPT_L)** **0.352 (GPT_R)**	–	–	0.216 (GPT_R)	0.237 (GPT_R)	–
ILF_L	**−0.376 (TMT-A)** **−0.309 (GPT_L)** **−0.340 (GPT_R)**	**0.311 (TMT-A)** 0.254 (GPT_L) **0.289 (GPT_R)**	**0.357 (TMT-A)** **0.290 (GPT_L)** **0.330 (GPT_R)**	–	−0.226 (GPT_R)	0.226 (GPT_L)	–	0.242 (GPT_L)
ILF_R	**−0.370 (TMT-A)** **−0.314 (GPT_L)** **−0.349 (GPT_R)**	**0.291 (TMT-A**) 0.226 (GPT_L) 0.276 (GPT_R)	**0.343 (TMT-A)** 0.276 (GPT_L) **0.321 (GPT_R)**	–	–	–	–	–
SLF_L	**−0.308 (TMT-A)** **−0.283 (GPT_L)** **−0.328 (GPT_R)**	0.245 (TMT-A) 0.256 (GPT_R)	**0.291 (TMT-A)** 0.232 (GPT_L) **0.300 (GPT_R)**	–	–	–	–	−0.227 (goRT)
SLF_R	**−0.298 (TMT-A)** −0.270 (GPT_L) −0.283 (GPT_R)	0.248 (TMT-A)	**0.290 (TMT-A)** 0.225 (GPT_L) 0.252 (GPT_R)	–	–	–	–	–
UF_L	−**0.342 (TMT-A)** **−0.321 (GPT_L)** **−0.322 (GPT_R)**	**0.296 (TMT-A)** 0.240 (GPT_L) 0.261 (GPT_R)	**0.335 (TMT-A)** **0.289 (GPT_L)** **0.305 (GPT_R)**	–	**−0.344 (GPT_R)**	–	0.225 (GPT_R)	–
UF_R	**−0.281 (TMT-A)** −0.258 (GPT_L) −0.253 (GPT_R)	**0.291 (TMT-A)** 0.199 (GPT_L) 0.225 (GPT_R)	**0.315 (TMT-A)** 0.242 (GPT_L) 0.259 (GPT_R)	–	−0.210 (GPT_R)	–	–	–
**Projection fiber**								
CST_L	**−0.293 (TMT-A)** −0.202 (GPT_R)	0.221 (TMT-A)	**0.302 (TMT-A)** 0.185 (GPT_R)	–	–	–	–	–
CST_R	−0.237 (TMT-A)	0.213 (TMT-A)	0.260 (TMT-A)	–	–	–	–	–
**Commissural fiber**								
Fmaj	**−0.379 (TMT-A)** **−0.393 (GPT_L)** **−0.428 (GPT_R)**	**0.286 (TMT-A)** 0.232 (GPT_L) **0.287 (GPT_R)**	**0.363 (TMT-A)** **0.341 (GPT_L)** **0.388 (GPT_R)**	–	−0.243 (GPT_R)	–	–	–
Fmin	**−0.430 (TMT-A)** **−0.400 (GPT_L)** **−0.355 (GPT_R)** −0.243 (goRT)	0.234 (TMT-A) 0.248 (GPT_L) 0.250 (GPT_R)	**0.349 (TMT-A)** **0.348 (GPT_L)** **0.323 (GPT_R)**	–	**−0.311 (GPT_R)**	–	0.269 (GPT_R)	–

*The ratio score’s formula: [(Measure_TP2_ − Measure_TP1_)/Measure_TP1_] × [2/(TP2 − TP1)]. The r value denoted in the table represents its p value passing the bootstrap criteria (i.e., upper and lower bound do not pass 0). r value marked in bold indicates its p value also passing the Bonferroni correction criteria (p < 0.0003). ‘–’ denotes non-significance.*

***Longitudinal*** result (***aging*** effect): Table 4 right columns show significant correlations between Δprocessing speed and ΔFA/MD/RD/AxD measures. Contrary to the cross-sectional result, there were relatively fewer significant correlations between Δprocessing speed and ΔDTI for the follow-up data. These include negative correlations between processing speed and nine tracts in FA, and positive correlations between processing speed and five tracts in MD, five tracts in RD, and four tracts in AxD (see Table 4 for details).

#### Common/Inhibition Component and Diffusion Tensor Imaging

***Cross-sectional*** result (***age*** effect): Table 5 left columns show significant correlations between the common/inhibition component and FA/MD/RD/AxD measures for the cross-sectional data (TP1). We can find there were many significant correlations between the common/inhibition component and DTI metrics, with FA being as negative correlations, and MD and RD being as positive correlations. Yet, AxD showed relatively fewer significant positive correlations in WM tracts (only in Fmin tract) with the common/inhibition component.

**TABLE 5:** The significant Pearson correlation *r* values for the common/inhibition component in relation to DTI measures (FA, MD, RD, and AxD) for cross-sectional and longitudinal ratio scores.

	Behavior (covariate: gender, edu, BDI-II)							
	Cross-sectional and common/inhibition				Longitudinal and common/inhibition			
	FA	MD	RD	AxD	FA	MD	RD	AxD
**Association fiber**								
ATR_L	–	0.217 (commonEF)	0.222 (commonEF)	–	–	–	–	–
ATR_R	–	–	0.223 (SSRT) 0.227 (commonEF)	–	–	–	–	–
CG_L	−0.264 (SSRT)	–	0.206 (SSRT)	–	–	–	–	–
CG_R	–	–	–	–	–	–	–	–
CH_L	–	–	–	–	–	–	–	–
CH_R	–	–	–	–	–	–	–	–
IFF_L	−0.255 (SSRT) −0.240 (commonEF)	0.216 (commonEF)	0.212 (SSRT) 0.239 (commonEF)	–	–	–	–	–
IFF_R	−0.252 (SSRT) −0.245 (commonEF)	–	0.211 (SSRT) 0.232 (commonEF)	–	–	–	–	–
ILF_L	−0.278 (SSRT) −**0.279 (commonEF)**	0.216 (commonEF)	0.222 (SSRT) 0.254 (commonEF)	–	–	–	–	–
ILF_R	−0.267 (SSRT) −0.267 (commonEF)	0.187 (commonEF)	0.203 (SSRT) 0.235 (commonEF)	–	–	–	–	–
SLF_L	−0.254 (SSRT) −0.217 (commonEF)	0.199 (commonEF)	0.206 (SSRT) 0.219 (commonEF)	–	–	–	–	–
SLF_R	−0.240 (SSRT)	0.200 (commonEF)	0.212 (SSRT) 0.222 (commonEF)	–	–	–	–	–
UF_L	–	–	0.213 (commonEF)	–	–	–	–	–
UF_R	−0.249 (SSRT)	–	0.211 (commonEF)	–	–	–	–	–
**Projection fiber**								
CST_L	–	–	–	–	–	–	–	–
CST_R	–	–	–	–	–	–	–	–
**Commissural fiber**								
Fmaj	−0.210 (SSRT) −0.221 (commonEF)	–	0.203 (commonEF)	–	–	–	–	–
Fmin	**−0.319 (SSRT)** −0.278 (commonEF)	–	0.225 (SSRT) 0.220 (commonEF)	−0.172 (SSRT)	–	–	–	–

*The ratio score’s formula: [(Measure_TP2_ − Measure_TP1_)/Measure_TP1_] × [2/(TP2 − TP1)]. The r value denoted in the table represents its p value passing the bootstrap criteria (i.e., upper and lower bound do not pass 0). r value marked in bold indicates its p value also passing the Bonferroni correction criteria (p < 0.0003). ‘–’ denotes non-significance.*

***Longitudinal*** result (***aging*** effect): Table 5 right columns show significant correlations between the Δcommon/inhibition component and percentage changes in DTI measures. Contrary to the cross-sectional result, there were no significant correlations between the Δcommon/inhibition and ΔDTI measures for the follow-up data.

#### Shifting Component and Diffusion Tensor Imaging

***Cross-sectional*** result (***age*** effect): Table 6 left columns show significant correlations between the shifting component and DTI measures for the cross-sectional data (TP1). We can find there were a few significant correlations between the shifting component and FA/MD/RD measures, with FA being as negative correlations, and MD/RD being as positive correlations. AxD showed relatively fewer significant positive correlations in WM tracts (only in ATR_R and ILF_L tracts) with the shifting component (see Table 6 for details).

**TABLE 6:** The significant Pearson correlation *r* values for the shifting component in relation to DTI measures (FA, MD, RD, and AxD) for cross-sectional and longitudinal ratio scores.

	Behavior (covariate: gender, edu, BDI-II)							
	Cross-sectional and shifting				Longitudinal and shifting			
	FA	MD	RD	AxD	FA	MD	RD	AxD
**Association fiber**								
ATR_L	–	0.226 (TMT-B)	0.223 (TMT-B)	–	–	–	–	–
ATR_R	–	0.214 (TMT-B)	0.194 (TMT-B)	0.233 (TMT-B)	−0.176 (non-infSWI)	–	–	–
CG_L	–	–	–	–	0.164 (SWI)	−0.122 (SWI)	–	–
CG_R	–	–	–	–	–	–	–	–
CH_L	–	–	0.200 (non-infSWI)	–	–	–	0.214 (non-infSWI)	–
CH_R	–	–	–	–	–	–	–	–
IFF_L	−0.214 (non-iSWI)	0.216 (TMT-B) –	0.230 (non-iSWI)	–	–	–	–	–
IFF_R	−0.223 (non-iSWI)	0.195 (TMT-B)	0.221 (non-iSWI)	–	–	–	–	–
ILF_L	−0.267 (non-infSWI)	0.201 (TMT-B) 0.213 (non-infSWI)	0.251 (non-infSWI)	0.208 (TMT-B)	–	–	–	–
ILF_R	−0.260 (non-infSWI)	0.176 (TMT-B) 0.213 (non-infSWI)	0.255 (non-infSWI)	–	–	–	–	–
SLF_L	–	–	–	–	–	–	–	–
SLF_R	–	–	–	–	–	–	–	−0.203 (TMT-B)
UF_L	–	–	0.207 (non-infSWI)	–	–	–	–	–
UF_R	–	–	–	–	–	–	–	–
**Projection fiber**								
CST_L	–	–	–	–	–	–	–	–
CST_R	–	–	–	–	–	–	–	–
**Commissural fiber**								
Fmaj	−0.260 (non-iSWI) −0.190 (TMT-B)	0.216 (non-iSWI) 0.186 (TMT-B)	0.257 (non-iSWI)	–	–	0.166 (SWI)	–	0.163 (SWI)
Fmin	−0.208 (TMT-B)	–	–	–	–	0.166 (SWI)	–	–

*The ratio score’s formula: [(Measure_TP2_ − Measure_TP1_)/Measure_TP1_] × [2/(TP2 − TP1)]. The r value denoted in the table represents its p value passing the bootstrap criteria (i.e., upper and lower bound do not pass 0). r value marked in bold indicates its p value also passing the Bonferroni correction criteria (p < 0.0003). ‘–’ denotes non-significance.*

***Longitudinal*** result (***aging*** effect): Table 6 right columns show significant correlations between the Δshifting component and ΔFA/MD/RD/AxD. Contrary to the cross-sectional result, there were much fewer significant correlations between Δshifting and ΔFA/MD/RD/AxD in WM tracts (**FA:** ATR_R, CG_L; **MD:** CG_L, Fmaj, Fmin; **RD:** CH_L; **AxD:** SLF_R, Fmaj) for the follow-up data.

#### Working Memory/Updating Component and Diffusion Tensor Imaging

***Cross-sectional*** result (***age*** effect): Table 7 left columns show significant correlations between the working memory/updating component and DTI measures for the cross-sectional data (TP1). We can find there were a few significant correlations between the working memory/updating component and DTI measures, with FA being as negative correlations, and MD/RD/AxD being as positive correlations (see Table 7 for details).

**TABLE 7:** The significant Pearson correlation *r* values for the working memory/updating component and DTI measures (FA, MD, RD, and AxD for cross-sectional and longitudinal ratio scores.

	Behavior (covariate: gender, edu, BDI-II)							
	Cross-sectional and working memory/updating				Longitudinal and working memory/updating			
	FA	MD	RD	AxD	FA	MD	RD	AxD
**Association fiber**								
ATR_L	–	0.206 (2back)	0.209 (2back)	–	–	–	–	–
ATR_R	–	0.215 (2back)	0.224 (2back)	0.175 (2back)	–	–	–	–
CG_L	–	–	–	–	–	–	–	–
CG_R	–	–	–	0.206 (updating)	–	–	–	0.193 (1back)
CH_L	–	–	–	–	−0.183 (MIX) −0.256 (2back)	–	–	–
CH_R	–	–	–	–	−**0.372 (2back)**	–	–	–
IFF_L	−0.194 (2back)	0.238 (2back) 0.198 (updating)	0.236 (2back) 0.175 (updating)	0.182 (2back) 0.198 (updating)	–	–	–	–
IFF_R	–	0.214 (2back)	0.211 (2back)	–	–	–	–	–
ILF_L	−0.208 (2back)	0.213 (2back)	0.223 (2back)	–	–	–	–	–
ILF_R	–	–	–	−0.202 (MIX)	−0.210 (2back)	–	–	–
SLF_L	–	–	–	–	–	–	–	–
SLF_R	–	–	–	–	–	–	–	–
UF_L	–	–	–	–	–	–	–	–
UF_R	–	–	–	–	–	–	–	–
**Projection fiber**								
CST_L	–	–	–	–	–	–	0.191 (1back *d*′)	–
CST_R	–	–	–	–	–	–	–	–
**Commissural fiber**								
Fmaj	−0.188 (2back)	–	0.192 (2back)	–	–	–	–	0.177 (2back)
Fmin	−0.227 (2back)	–	0.199 (2back)	–	–	–	–	–

*The ratio score’s formula: [(Measure_TP2_ − Measure_TP1_)/Measure_TP1_] × [2/(TP2 − TP1)]. The r value denoted in the table represents its p value passing the bootstrap criteria (i.e., upper and lower bound do not pass 0). r value marked in bold indicates its p value also passing the Bonferroni correction criteria (p < 0.0003). ‘–’ denotes non-significance.*

***Longitudinal*** result (***aging*** effect): Table 7 right columns show significant correlations between the Δworking memory/updating component and percentage changes in DTI measures. Contrary to the cross-sectional result, there were much fewer significant correlations between Δworking memory/updating and ΔFA/MD/RD/AxD in WM tracts (**FA:** CH_L/R, ILF_R; **RD:** CST_L; **AxD:** CG_R, Fmaj) for the follow-up data.

### Mediation Model Among Age (Timepoint 1), Diffusion Tensor Imaging Changes, and Processing Speed Changes

**FIGURE 2:**
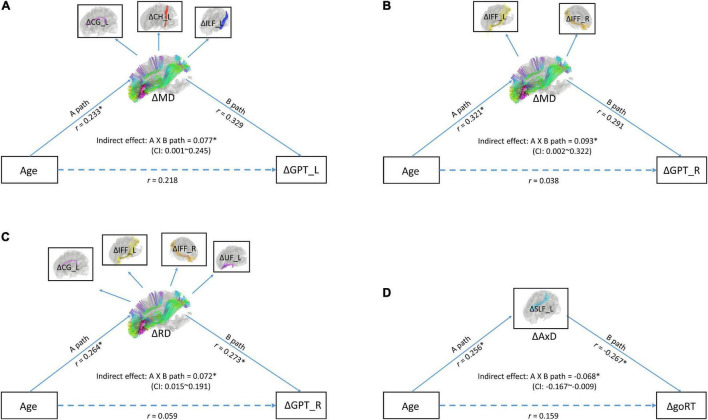
**(A)** A mediation model with latent variables for age (at time point 1), ΔMD (changes in mean diffusivity), and ΔGPT_L (changes in processing speed). **(B)** A mediation model with latent variables for age (at time point 1), ΔMD (changes in mean diffusivity), and GPT_R (changes in processing speed). **(C)** A mediation model with age (at time point 1), ΔRD, and GPT_R. **(D)** A mediation model with age (at time point 1), ΔAxD, and goRT (changes in processing speed). In these models, observed variables (shown in boxes) serve as independent variables and indicators of latent variables (shown in ellipses). Indirect effect between age and mixing cost was tested by the bootstrap method. Upper and lower bound of the 95% confidence interval was marked in the center of the model. GPT_L, Grooved Pegboard Test with the left hand; GPT_R, Grooved Pegboard Test with the right hand.

To directly test the idea if changes in MD (ΔMD), RD (ΔRD), and/or AxD (ΔAxD) mediated the relationship between age (at TP1) and changes in cognitive performance, especially ***processing speed***, we ran a series of mediation models. The results of model fittings showed that two models on ΔMD yielded good model fits. One model showed an indirect effect between age (at TP1) and changes in the neuropsychological task of GPT_L (an index of processing speed) with the latent variable of changes in MD (ΔMD) in WM tracts of CG_L, CH_L, and ILF_L as mediators (RMSEA < 0.06; chi square *p* = 0.188; CFI = 0.97) (see Figure 2A). The other model showed an indirect effect between age (at TP1) and changes in the neuropsychological task of GPT_R (an index of processing speed) with the latent variable of changes in MD (ΔMD) in WM tracts of IFF_L/R (RMSEA < 0.001; chi square *p* = 0.931; CFI = 1.00) (see Figure 2B). In addition, there was one good model fit on ΔRD showing that the latent variable of changes in WM tracts of CG_L, IFF_L/R, and UF_L mediated the relationship between age and the neuropsychological task of GPT_R (an index of processing speed) (RMSEA < 0.001; chi square *p* = 0.977; CFI = 1.00). A fourth good model fit is on ΔAxD, showing that the changes in AxD (ΔAxD) in WM tract of SLF_L mediated the relationship between age and changes in go RT (an index of processing speed).

## Discussion

This study aimed to provide longitudinal data to compare with cross-sectional data, in which we were interested in examining if age-related differences (cross-sectional) in WM tracts and cognitive control functions can be also observed in age-related changes data with a follow-up interval of 2 years (longitudinal).

Regarding the first issue of the relationship between age and cognitive control function (including processing speed) cross-sectionally and longitudinally, we observed that while there were several significant age-related differences in processing speed, common (inhibition), shifting, and working memory updating, there were no significant correlations for age-related changes in cognitive control functions. Only changes in processing speed from TP1 to TP2 reflected on GPT_L were significantly related to age (at TP1). The results suggest that many significant correlations between age and cognitive control functions originally shown on cross-sectional data no longer exist on the longitudinal data, except that the relationship with age for ***processing speed*** was still retained even in the longitudinal data.

The current findings bring our attention in the fact that although we could observe age-related cognitive control deficits which are typically reported in most literature using cross-sectional approach (e.g., less working memory capacity – Salthouse, 1994; West, 1996; Grady and Craik, 2000; a deficit in inhibitory processing – Hasher and Zacks, 1988; Kramer et al., 1994; a lack of cognitive flexibility – Kramer et al., 1999; Mayr, 2001), when we followed up these individuals across the adult lifespan over an average of 2 years, we did not see many differences (except ***processing speed*** reflected on the left hand’s movement) in their changing trajectories. Literature has also indicated a similar finding in that age did not significantly correlate with working memory changes over a period of 2 years (Charlton et al., 2010; Lövdén et al., 2014). Therefore, we should not overlook the possibility that ***aging*** processing in cognitive control functions likewise occurs for younger adults. ***That is, the aging process in cognitive control functions may not be older-age specific, but more generally occur across lifespan.***

Regarding the second issue of the relationship between age and DTI measures (FA, MD, RD, AxD), we observed that FA indexes in many WM tracts at TP1 were sensitive to ***age*** effect (cross-sectional data: all tracts except CG_R, CH_L, CH_R, and CST_R). In particular, Fmin has the strongest age effect reflected on FA, which is in line with the findings in the literature either using a longitudinal or cross-sectional approach (e.g., longitudinal approach: Barrick et al., 2010; cross-sectional approach: Salat et al., 1997; Vernooij et al., 2008; Sullivan et al., 2010; Jolly et al., 2016; Zavaliangos-Petropulu et al., 2019; Hsieh et al., 2020). Conversely, the FA index’s changes from TP1 to TP2 were **much less** correlated to the ***aging*** effect (i.e., follow-up data). The result suggests that FA might ***not*** be a robust index when examining the ***aging*** effect. On the other hand, both MD and RD at TP1 and their changes from TP1 to TP2 showed relatively similar relationships with age (except CST_L/R, Fmaj, and Fmin tracts)^2^ Although AxD showed a different pattern from other DTI measures in relation to age on the cross-sectional data (e.g., they were more non-linearly correlated with age compared to other DTI metrics), its changes somehow showed similar patterns as MD and RD changes in the follow-up results, in which several WM tracts were significantly correlated with aging (except Fmaj and Fmin tracts). Although previously based on the cross-sectional data, even our lab also claimed that FA might play a more important role than MD, RD, or AxD in reflecting the age effect (Hsieh et al., 2020), the current results based on both cross-sectional and longitudinal data conversely suggest that MD, RD, and AxD indexes in WM tracts might be more representative than FA when examining both ***age*** and ***aging*** effects with DTI (note: AxD yielded more non-linear correlations with age cross-sectionally). FA is a ratio value reflecting the directional consistency of diffusion, with higher values indicating that diffusion within a voxel is primarily restricted to one direction (O’Donnell and Westin, 2011), conversely, MD refers to the average amount of diffusion occurring within an image voxel, RD is calculated as the amount of diffusion perpendicular to the main directional axis of fibers, and AxD refers to the magnitude of diffusivity parallel to fiber tracts. Early studies of optic nerve fiber damage (Song et al., 2003, 2005) indicated that increases in RD values are associated with axonal demyelination (Wheeler-Kingshott and Cercignani, 2009), whereas lower AxD may reflect axonal injury, reduced axonal caliber, or less coherent orientation axons. Literature has shown a negative correlation between age and FA, suggesting that in older individuals the diffusion tensor is less fractionally anisotropic than in relatively younger individuals (e.g., a cross-sectional study by Boekel and Hsieh, 2018). In addition, literature using a cross-sectional approach also often report a positive correlation between MD, RD, or AxD and age (e.g., Sullivan et al., 2001; Pfefferbaum and Sullivan, 2003; Abe et al., 2008; Westlye et al., 2009; Burzynska et al., 2010; Lebel et al., 2012). Since the current longitudinal data showed that MD, RD, and AxD changes are more closely related than FA changes to the aging process, we would suggest that axonal demyelination and/or axonal degeneration is a more robust phenomenon for observing intra-individual aging processes.

When we further compared age-related declines in different WM tracts, we found association fibers of CG_R and CH_L/R were less correlated to both ***age*** and ***aging*** effects when compared to other WM tracts. In addition, ATR_L/R, and Fmin tracts across FA/MD/RD/AxD appeared to be most correlated to the ***age*** effect (cross-sectional data), in which the findings are consistent with the suggestion of frontal and callosal areas being most affected by the ***age*** effect (e.g., cross-sectional studies: Zavaliangos-Petropulu et al., 2019; Hsieh et al., 2020; Behler et al., 2021).

However, the current longitudinal data showed that the commissural fiber of Fmin no longer yielded an ***aging*** effect, despite its strong relationship with the ***age*** effect cross-sectionally. On the other hand, although the frontal association fibers of ATR_L/R remain to be correlated with ***agin***g, they were only present in MD/RD/AxD, but no in FA. ***Therefore, combining the cross-sectional and longitudinal results in the current study, we suggest that ATR tracts in MD/RD/AxD are the most representative when examining both age and aging effects.***

Turning to the third issue of the association between cognitive control functions (including processing speed) and DTI measures, we observed that ***processing speed*** and cognitive control functions (including common/inhibition, shifting, and working memory/updating) were related to DTI measures cross-sectionally, with ***processing speed*** showing the most correlations with WM tracts in FA/MD/RD. Literature using a cross-sectional approach has also shown that age-related WM decline may contribute to age-related cognitive control declines. For example, some studies have explored age-dependent relationships between white matter integrity and composite measures of cognitive control function (Kennedy and Raz, 2009; Vernooij et al., 2009; Zahr et al., 2009). Furthermore, some researchers have found that frontoparietal WM differences are linked to age-related differences in task-switching performance (Madden et al., 2009; Gold et al., 2010). Perry et al. (2009) using a cross-sectional approach also observed that advancing age was associated with declines in task-set shifting performance and with decreased FA in corpus callosum and in association tracts that connect the frontal cortex to more posterior brain regions. A more recent DTI study using a cross-sectional approach led by Karayanidis also demonstrated the role of WM microstructure in age-related cognitive decline (Jolly et al., 2013). Bucur et al. (2008) also using a cross-sectional approach observed that declines in FA in the pericallosal frontal region and in the genu of the corpus callosum, but not in other regions, mediated the relationship between perceptual speed and episodic retrieval reaction time. Hence, they suggested that WM integrity in prefrontal regions is one mechanism underlying the relationship between individual differences in perceptual speed and episodic retrieval (Bucur et al., 2008). Nevertheless, most of these studies cited above are cross-sectional results.

However, when we examined the longitudinal data in the current study, we observed that changes in ***processing speed*** as compared to changes in other cognitive control performance were more correlated to the changes in all DTI indexes. Therefore, ***processing speed*** appeared to be the function that was mostly related to WM integrity both cross-sectionally and longitudinally. Processing speed is a basic cognitive or brain process that subserves many other higher-order cognitive domains. One possibility is that over a short period of follow up, changes in processing speed would be more apparent than other cognitive control functions, thus would be easier to be observed to be related to changes in WM integrity. Future research with a longer period of follow up is needed to clarify the speculation.

Finally, to provide a more direct evidence in showing if changes in DTI mediated age-related changes in ***processing speed***, we tested a series of mediation models, and the result showed that four models yielded good model fits, in which ΔRD, ΔMD, and ΔAxD mediated age-related changes in ***processing speed***. Therefore, converging the regression results and mediation models, we could conclude that MD, RD, and AxD appear to be the most representative DTI measures to reveal age-related changes in ***processing speed***.

Some final remarks are worth mentioning before closing. First, in the current study, only a period of 1–2 years follow-up interval was performed, it is likely that the period is too short to be sensitive enough to reveal the aging process. For example, in the literature, Charlton et al. (2010) and Lövdén et al. (2014) using a follow-up approach likewise did not find an aging effect on cognitive changes, but Vonk et al. (2020) found a linear aging effect in working memory and cognitive speed. The discrepant findings among these studies are likely due to their differences in the length of the follow-up interval. The follow-up interval between the two timepoints in Vonk et al.’s (2020) research was 4∼8 years, whereas in the other two research (i.e., Charlton et al., 2010; Lövdén et al., 2014), the age interval was about 2 years which is similar to the current study. Therefore, future studies with a longer follow-up interval or more times of follow-up are warranted. Second, while we examined the aging effect in relation to the changes in DTI from TP1 to TP2, we observed very few and even paradoxically positive correlations for three WM tracts (i.e., CH_L/R, CST_L) in FA. The finding was the opposite to the cross-sectional results reported by the prior and current research. One possibility is that the reduction rate for FA with aging was not as prominent as one could expect from other DTI indicators, and because this study only followed 1.77 years on average, thus very few significant changes could be observed for FA. However, why these three tracts (CH_L/R, CST_L) showed FA increases rather than decreases with aging remain puzzled. We suspected that individual differences might contribute to the paradoxical findings. One longitudinal study with 1-year follow up comparing healthy control and Alzheimer’s disease also found that healthy controls did not demonstrate FA changes in the hippocampal cingulum (i.e., CH) as were observed in those with Alzheimer’s disease (Mayo et al., 2017). The authors thus concluded that changes in microstructural integrity for the hippocampal cingulum over short time intervals (i.e., 1 year) may more specifically reflect ongoing degenerative processes due to Alzheimer’s disease. Since the participants in the current study were all healthy controls, some of them might be even more reserved than middle-aged or young adults. Future research with a longer follow-up period and considering individual differences is needed to clarify the issue. Third, the participants’ age range in this study covered a wide range of 20 to 80 years, it may be worth considering sub-groups of different ages rather than treat all participants as a single group (e.g., see Yap et al., 2013 and Lebel et al., 2019 for reviews of DTI findings related to development). Future studies with more numbers of participants are encouraged to split into subgroups with smaller age ranges (e.g., every 10 years old per group). Fourth, this study did not analyze myelin water fraction (MWF) which should not be overlooked and should be analyzed complementary to the conventional DTI metrics. A recent study (Kiely et al., 2022) has indicated that although DTI metrics (e.g., RD and FA – indices of myelin content) might be related to the age effect, they could not serve as specific metrics to myelin, so that further studies using more specific myelin measure, such as MWF relaxometry, are required. Fifth, in this study, we used the TBSS processing pipeline in the FSL, which was developed to reduce the effects of local mis-registrations by projecting all FA voxels onto the nearest location on a “skeleton” approximating WM tract centers (Smith et al., 2006). Some studies however pointed out that TBSS was designed to compensate for local registration errors, which might in return cause many more limitations due to over compromises (see Zalesky, 2011; Schwarz et al., 2014, for more examples). Despite the limitations outlined, TBSS remains a popular method for DTI analyses.

To conclude, the current results warrant the importance of longitudinal research for aging studies to elucidate actual *aging* processes on cognitive control function. More critically, the current results provide new insights to which indicator of WM integrity and which type of cognitive changes are most representative (i.e., potentially to be effective neuroimaging biomarkers) to reflect intra-individual cognitive aging processes.

## Data Availability Statement

The raw data supporting the conclusions of this article will be made available by the authors, without undue reservation.

## Ethics Statement

The studies involving human participants were reviewed and approved by Research Ethics Committee of the National Cheng Kung University, Tainan, Taiwan (Contract No. 104–004). The patients/participants provided their written informed consent to participate in this study. Written informed consent was obtained from the individual(s) for the publication of any potentially identifiable images or data included in this article.

## Author Contributions

SH contributed to the grant resources, the design of the experiments, data processing, and preparation of the manuscript. M-HY contributed to the data collection and analysis, and preparation of figures and tables. Both authors contributed to the article and approved the submitted version.

## Conflict of Interest

The authors declare that the research was conducted in the absence of any commercial or financial relationships that could be construed as a potential conflict of interest.

## Publisher’s Note

All claims expressed in this article are solely those of the authors and do not necessarily represent those of their affiliated organizations, or those of the publisher, the editors and the reviewers. Any product that may be evaluated in this article, or claim that may be made by its manufacturer, is not guaranteed or endorsed by the publisher.

## Footnotes

1
www.fmrib.ox.ac.uk/fsl

2 In the current study, we further found that there was a non-linear aging trajectory for MD and RD showing a knob transition occurred around 50 years old for cross-sectional data at TP1 (see Figure 2).
