# Supplementary material for: Potential Diffusion Tensor Imaging Biomarkers for Elucidating Intra-Individual Age-Related Changes in Cognitive Control and Processing Speed
Source: Front Aging Neurosci. 2022 Apr 26;14:850655. doi: 10.3389/fnagi.2022.850655 (PMC9087335; doi:10.3389/fnagi.2022.850655)
Supplement: Supplementary file 1 [file Data_Sheet_1.PDF]

Supplementary Table S1: Means and paired t-tests between the two time points (TP1 vs. TP2) for cognitive control tasks

	TP1	TP2	paired t-test (*p*)
TMT-A	30.611(±13.415)	26.085(±11.357)	0.000
TMT-B	58.596(±27.443)	50.097(±21.406)	0.000
GPT_L	78.816(±16.021)	80.738(±20.743)	0.081
GPT_R	74.452(±14.041)	74.127(±16.531)	0.752
SSRT	253.362(±104.065)	239.030(±86.716)	0.085
goRT	642.761(±149.390)	600.216(±119.059)	0.000
SWIcost	76.758(±112.930)	69.789(±90.202)	0.537
noninfSWIcost	240.516(±127.227)	239.034(±134.162)	0.900
MIXcost	133.288(±142.660)	83.479(±129.900)	0.000
2back d'	1.904(±0.868)	2.108(±0.907)	0.002
1back d'	2.973(±0.765)	3.207(±0.623)	0.001
commonEF	0.000(±0.634)	0.001(±0.651)	0.997
Shifting	-0.001(±0.995)	0.000(±0.996)	0.987
Updating	0.001(±0.995)	0.004(±0.995)	0.973

Supplementary Table S2: Means and paired t-tests between the two time points (TP1 vs. TP2) for white matter integrity (tract-of-interest)

	FA			$MD*10^{-3}$			$RD*10^{-3}$			$AxD*10^{-3}$		
	TP1	TP2	(p)	TP1	TP2	(p)	TP1	TP2	(p)	TP1	TP2	(p)
**Association fiber**												
ATR_L	0.438(±0.017)	0.437(±0.017)	0.003	0.788(±0.031)	0.794(±0.035)	0.000	0.587(±0.033)	0.592(±0.036)	0.000	1.191(±0.031)	1.198(±0.036)	0.000
ATR_R	0.432(±0.016)	0.432(±0.016)	0.804	0.796(±0.033)	0.803(±0.035)	0.000	0.596(±0.033)	0.601(±0.036)	0.000	1.195(±0.034)	1.205(±0.038)	0.000
CG_L	0.507(±0.023)	0.504(±0.023)	0.000	0.749(±0.026)	0.754(±0.026)	0.000	0.511(±0.030)	0.516(±0.030)	0.000	1.226(±0.030)	1.230(±0.030)	0.000
CG_R	0.486(±0.024)	0.480(±0.023)	0.000	0.745(±0.022)	0.751(±0.022)	0.000	0.523(±0.025)	0.530(±0.026)	0.000	1.189(±0.034)	1.194(±0.033)	0.001
CH_L	0.406(±0.024)	0.407(±0.023)	0.277	0.793(±0.032)	0.799(±0.032)	0.001	0.605(±0.034)	0.609(±0.033)	0.005	1.171(±0.040)	1.180(±0.042)	0.001
CH_R	0.424(±0.026)	0.427(±0.025)	0.006	0.796(±0.035)	0.792(±0.029)	0.024	0.599(±0.037)	0.594(±0.031)	0.006	1.190(±0.042)	1.188(±0.041)	0.453
IFF_L	0.452(±0.021)	0.450(±0.021)	0.008	0.768(±0.025)	0.774(±0.027)	0.000	0.560(±0.029)	0.565(±0.031)	0.000	1.184(±0.025)	1.190(±0.027)	0.000
IFF_R	0.464(±0.021)	0.461(±0.021)	0.000	0.775(±0.027)	0.780(±0.029)	0.000	0.558(±0.031)	0.564(±0.032)	0.000	1.208(±0.027)	1.213(±0.028)	0.000
ILF_L	0.445(±0.019)	0.445(±0.020)	0.769	0.784(±0.026)	0.787(±0.028)	0.000	0.574(±0.029)	0.576(±0.030)	0.012	1.204(±0.028)	1.208(±0.030)	0.001
ILF_R	0.433(±0.019)	0.432(±0.019)	0.379	0.779(±0.026)	0.782(±0.027)	0.001	0.579(±0.029)	0.582(±0.030)	0.003	1.180(±0.027)	1.182(±0.028)	0.052
SLF_L	0.436(±0.018)	0.437(±0.018)	0.383	0.739(±0.024)	0.741(±0.025)	0.036	0.552(±0.026)	0.553(±0.027)	0.209	1.115(±0.025)	1.116(±0.027)	0.143
SLF_R	0.439(±0.017)	0.438(±0.017)	0.432	0.739(±0.023)	0.742(±0.024)	0.003	0.551(±0.024)	0.553(±0.026)	0.021	1.116(±0.026)	1.118(±0.027)	0.024
UF_L	0.421(±0.021)	0.419(±0.021)	0.003	0.757(±0.025)	0.762(±0.026)	0.000	0.571(±0.029)	0.576(±0.030)	0.000	1.128(±0.026)	1.135(±0.027)	0.000
UF_R	0.423(±0.021)	0.419(±0.020)	0.000	0.774(±0.027)	0.781(±0.028)	0.000	0.579(±0.029)	0.587(±0.030)	0.000	1.161(±0.030)	1.169(±0.030)	0.000
**Projection fiber**												

CST_L	0.563(±0.017)	0.562(±0.018)	0.014	0.725(±0.016)	0.726(±0.017)	0.596	0.464(±0.019)	0.465(±0.021)	0.241	1.248(±0.026)	1.249(±0.026)	0.555
CST_R	0.553(±0.016)	0.552(±0.017)	0.374	0.730(±0.016)	0.731(±0.017)	0.376	0.474(±0.019)	0.475(±0.020)	0.279	1.241(±0.025)	1.243(±0.025)	0.242
**Commissural fiber**												
Fmaj	0.530(±0.022)	0.528(±0.022)	0.000	0.763(±0.025)	0.768(±0.027)	0.000	0.497(±0.030)	0.501(±0.031)	0.000	1.295(±0.030)	1.304(±0.031)	0.000
Fmin	0.496(±0.025)	0.494(±0.025)	0.000	0.762(±0.030)	0.771(±0.031)	0.000	0.525(±0.034)	0.533(±0.036)	0.000	1.238(±0.033)	1.246(±0.030)	0.000
